# Time to Diagnosis and Treatment of Lyme Disease by Patient Race

**DOI:** 10.1001/jamanetworkopen.2023.47184

**Published:** 2023-12-12

**Authors:** Samuel J. Starke, Alison W. Rebman, John Miller, Ting Yang, John N. Aucott

**Affiliations:** 1 Department of Medicine, Johns Hopkins University School of Medicine, Baltimore, Maryland; 2 Lyme Disease Research Center, Division of Rheumatology, Department of Medicine, Johns Hopkins University School of Medicine, Baltimore, Maryland

## Abstract

This cross-sectional study compares stages at which Lyme disease was diagnosed by race in a specialty clinic in the US.

## Introduction

Early diagnosis of Lyme disease (LD) requires prompt recognition of the erythema migrans (EM) rash and/or viral-like symptoms. Untreated infection can progress to disseminated disease, which is associated with more significant, prolonged morbidity.^1^ Therefore, identifying and addressing gaps in early diagnosis are paramount; however, large, clinic-based studies have been lacking.

## Methods

In this cross-sectional study, we analyzed data from patients with suspected untreated or posttreatment LD seen either for clinical care or research participation at a specialty clinic in suburban Maryland. All patients self-reported their demographic information, and clinical information was either recorded at diagnosis or abstracted from existing external medical records. The Johns Hopkins University School of Medicine institutional review board approved these studies. Research participants received a stipend and provided written informed consent, while consent was waived for abstraction of clinic records. We followed the Strengthening the Reporting of Observational Studies in Epidemiology (STROBE) reporting guideline for cross-sectional studies.

We created the following initial LD presentations: (1) EM only, with no objective evidence of disseminated disease; (2) disseminated neurologic, cardiac, or joint disease, defined as having objective evidence and without considering prior EM; or (3) symptoms only, defined as patient-reported suspected LD without objective signs. We examined the racial distribution across groups using χ^2^ or Fisher exact tests. To test the hypothesis that Black patients have higher rates of disseminated disease and lower rates of objective findings, we fit multivariable logistic regression models with robust standard errors. We conducted 1-sided Mann-Whitney tests to test the hypothesis that Black patients would have a longer time to appropriate treatment. Statistical analyses were performed using R version 4.3.1 (R Foundation for Statistical Computing) and SAS version 9.4 (SAS Institute Inc), and the graph was generated using Prism version 10.0.2 (GraphPad). *P* < .05 was considered significant—all group comparison tests were 2-sided and the test for time to treatment by race was 1-sided. Additional methods details are available in eMethods in Supplement 1.

## Results

**Figure 1.**
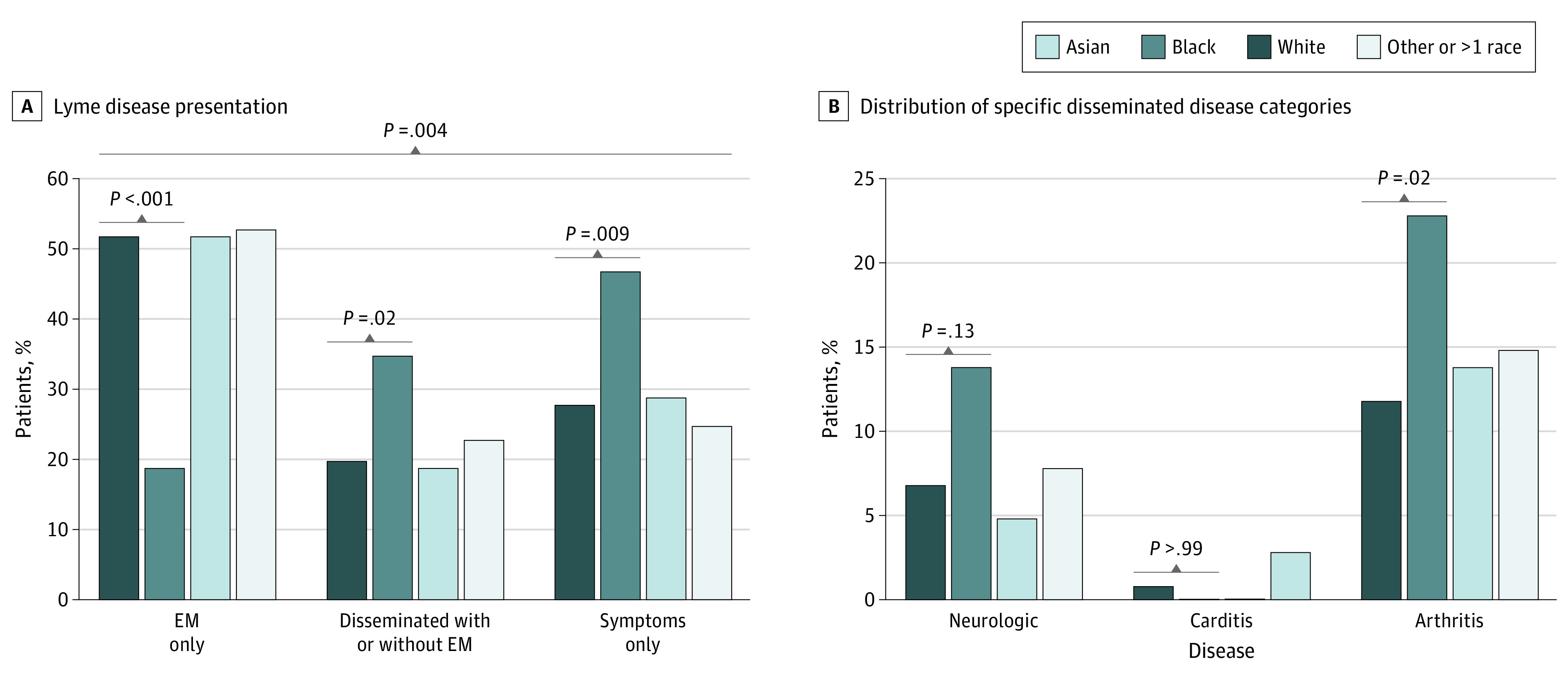
Distribution of Initial Lyme Disease Presentation by Race Patients (1395 total) self-identified race based on options defined in the electronic medical record, including 21 Asian, 43 Black, 1291 White, and 40 patients reporting 1 or more race. Asian patients and patients self-identifying as other or more than one race are included; however, as differences were most marked among Black patients, subsequent analyses have focused on comparisons between Black and White patients. Although provided as response options, no patients identified solely as Native Hawaiian and Pacific Islander or American Indian and Alaskan Native. Panel B depicts the distribution of specific disseminated disease categories by race (283 patients). Disseminated disease categories are not mutually exclusive and a small number of patients presented with more than 1.

The final sample included 1395 patients, of which 703 (50.4%) were men with a median (IQR) age at LD of 48 (35-60) years. Figure 1 shows the distribution of initial LD presentation by race. Differences were most marked among Black patients; therefore, subsequent analyses focused on comparisons between Black and White patients (1334 patients total).

After controlling for age and gender, Black patients had 4.93 times the odds of disseminated disease (95% CI, 2.02-12.02; *P* < .001) compared with the EM only group (949 patients). Men independently also had higher odds of disseminated disease (OR, 1.61; 95% CI, 1.20-2.15; *P* = .001). Among 1325 patients, Black patients (OR, 2.07; 95% CI, 1.12-3.84; *P* = .02), women (OR, 1.39; 95% CI, 1.09-1.77; *P* = .007), and younger age (per 10 years: OR, 1.12; 95% CI, 1.04-1.20; *P* = .002) all independently had higher odds of being in the symptoms only group.

Overall, Black patients had a significantly longer median (IQR) time to appropriate antibiotic treatment (35 [6-119] days) compared with White patients (7 [0-70] days) (1259 patients; *P* = .01). This was significant among patients with EM (Black, 26 [6-36] days; White, 4 [0-14] days; *P* = .05) but not those with disseminated disease or symptoms only. Initial inappropriate antibiotics were found in 6 of 37 Black patients (16.2%) and 90 of 1165 White patients (7.7%) (*P* = .06).

## Discussion

**Figure 2.**
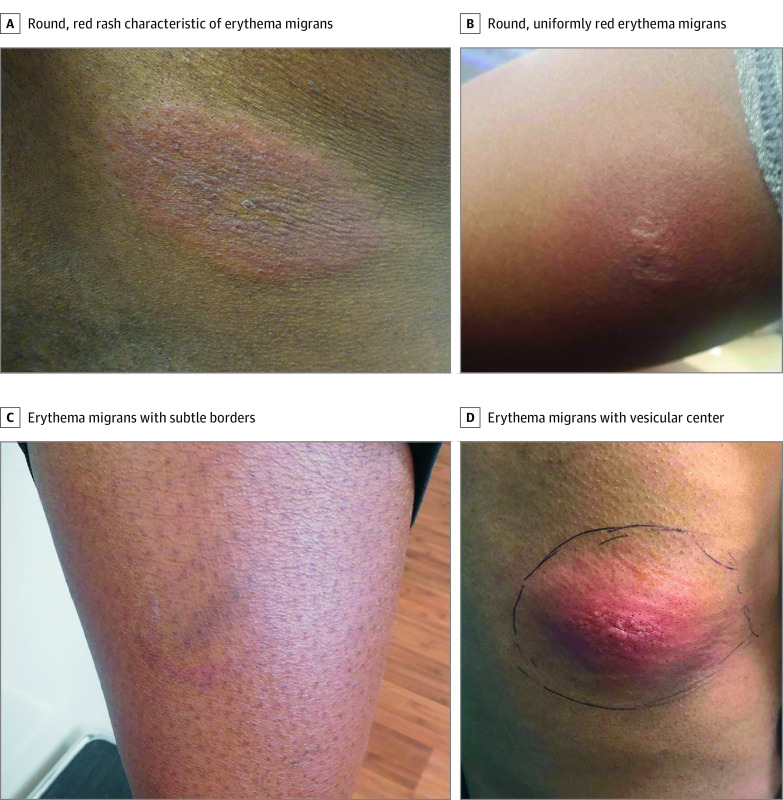
Erythema Migrans Rashes on Black Patients Example images of erythema migrans rashes with typical oval shape from patients in the study sample self-identifying as Black. The patients in panels B and D were initially misdiagnosed with an allergic reaction and cellulitis, respectively, but subsequently tested positive for Lyme disease. The patient in panel D was initially inappropriately treated with 7 days of cefalexin after appearance of their rash.

In one of the few large studies of racial differences in LD in an academic setting, we found that Black patients were more likely to be diagnosed with disseminated manifestations and experience longer time to appropriate treatment, consistent with prior surveillance and insurance claims studies.^2,3,4^ EM images on Black patients (Figure 2) are underrepresented in medical education materials,^5,6^ and gaps in health care access, racial discrimination, and implicit bias doubtlessly also contribute to EM underrecognition.

Our study is limited by the relatively small number of Black patients in our sample, and may have variable generalizability to non-US settings, or to patients seen outside a suburban, academic clinical setting. Additionally, we were unable to incorporate other factors that may affect access to health care in our analyses. Regardless, we identified several racial differences in the clinical presentation and treatment of LD. Efforts are needed to increase patient and clinician awareness to ensure equitable reductions in disease burden.

## Notes

Supplement 1.
eMethods

Supplement 2.
Data Sharing Statement
