# Supplementary material for: Time to Diagnosis and Treatment of Lyme Disease by Patient Race
Source: JAMA Netw Open. 2023 Dec 12;6(12):e2347184. doi: 10.1001/jamanetworkopen.2023.47184 (PMC10716730; doi:10.1001/jamanetworkopen.2023.47184)
Supplement: Supplement 1. — eMethods [file jamanetwopen-e2347184-s001.pdf]

Supplemental Online Content

Starke SJ, Rebman AW, Miller J, Yang T, Aucott JN. Time to diagnosis and treatment of Lyme disease by patient race. *JAMA Netw Open.* 2023;6(12):e2347184. doi:10.1001/jamanetworkopen.2023.47184

eMethods.

This supplemental material has been provided by the authors to give readers additional information about their work.

© 2023 Starke SJ et al. *JAMA Network Open.*

eMethods.

Patient Sample

In the current study, data were pooled from an initial convenience sample of 1,423 adult (≥ 18 years) patients seen at a specialty clinic in suburban Maryland for either clinical care (from 2015-2022) or research participation (from 2008-2022). We then excluded those patients missing race information (n=9, 0.6%) and those seen clinically where no signs, symptoms, or laboratory testing suggestive of LD could be found in medical record review (n=19, 1.3%), for a final sample of 1,395.

This final sample included data from three primary sources. The first was a longitudinal cohort study of patients with at least one visible, diagnostic erythema migrans rash at enrollment who self-referred or were recruited from primary or urgent care settings (n=381, 27.3%). Participants were excluded for several conditions associated with significant fatigue, immunosuppression, psychiatric disease, or autoimmune illness. Clinical information regarding initial Lyme disease presentation for these patients was recorded at the time of diagnosis and enrollment into the study. The second was a clinical case series of patients meeting a research case definition for post-treatment Lyme disease, with probable or confirmed prior Lyme disease verified by medical record review, and current functionally-impairing symptoms (n=286, 20.5%). These patients were primarily recruited from those seen at the clinic who met eligibility criteria and agreed to participate, and were excluded for the same list of conditions detailed above. Clinical information regarding initial Lyme disease presentation for these patients was abstracted from medical records at the time of initial Lyme disease diagnosis and treatment. Finally, a third source was a clinic chart review of patients seen solely for clinical care of Lyme disease, in which their initial presentation was also abstracted in the same way from their medical records (n=728, 52.2%).

In the current study, the merged sample across these sources was reviewed to ensure that any patients seen both for clinical care and research participation were not duplicated. The final sample therefore included 728 (52.2%) patients seen for clinical care only, and 667 (47.8%) seen as part of a research study.

Data Abstraction

© 2023 Starke SJ et al. *JAMA Network Open.*

For the 1,014 patients whose initial Lyme disease presentation information was abstracted from their medical record (the second and third sources), a standardized data abstraction instrument with explicitly defined variables was created by two of the authors (JNA and AWR), who also oversaw the abstraction process. The first series of abstractions (n=32, 3.2%) were performed by AWR as part of testing the validity of the instrument and training subsequent abstractors, one of whom performed n=10 (1%) of abstractions and the other performed the remaining n=972 (95.9%) of abstractions across several years. All abstractors were unaware of the current study's hypothesis at the time of abstraction, and were encouraged to raise questions and concerns as they arose. The resulting data were edited for internal consistency and missing information prior to any analyses being performed. Our data integrity efforts worked to minimize any instances of missing data. These data were collected and managed using REDCap (Research Electronic Data Capture) tools hosted at Johns Hopkins University.

Variables of Interest

The primary variables of interest in the current study included a) race, which was first described by the categories displayed in Figure 1 then treated as a binary variable (Black vs. white) in subsequent analyses that excluded other races, and b) Lyme disease initial presentation, as described in the manuscript as three categories. Age was abstracted as a continuous variable and descriptively summarized in its original one-year units. For an accurate and easier interpretation of the estimated odds ratio in the final adjusted model, age was rescaled to 10-year units by dividing the original continuous age variable by 10. Gender was assessed through self-report, with one patient identifying as neither a man or a woman.

Time to appropriate treatment was determined as the interval between the date of the patient's first self-reported new-onset sign or symptom of their Lyme disease and the date of initiation of the first appropriate antibiotic treatment for Lyme disease. Appropriate antibiotic treatment for Lyme disease was considered to be any of the following identified in the medical record as prescribed at the time of initial diagnosis: Doxycycline 100mg BID for at least 10 days, Tetracycline 500mg TID for at least 14 days, Amoxicillin 500mg TID for at least 14 days, Augmentin 875mg BID for at least 14 days, Ceftin 500mg BID for at least 14 days, and Ceftriaxone 2g Q24 for at least 14 days.

© 2023 Starke SJ et al. *JAMA Network Open.*

Statistical Methods

We performed two final multivariable logistic regression models after conducting initial univariate models to test for variable inclusion. The first tested ‘Disseminated Disease’ (compared to the ‘EM only’ group) as the outcome, while Black (vs. white) was the primary predictor of interest, and age and gender were included as potential confounders. The second tested the ‘Symptoms Only’ group (compared to both the ‘EM only’ and ‘Disseminated Disease’ groups combined) with the same primary predictors and potential confounders. Gender and age were included as potential confounders because prior studies have suggested differences in initial Lyme disease presentation by these two factors. P<.15 was used to determine variable inclusion in subsequent adjusted, multivariate models. Robust standard errors were used for all models.

No patients included in the final sample were missing clinical Lyme disease presentation category or race. We did not perform any imputation on missing data, we chose instead to omit patients with incomplete data from specific analyses based on the assumption that these would be missing completely at random (MCAR). In the analyses focused on comparisons between Black and white patients (n=1,334), missing data included the following: one individual identifying as neither a man or a woman was excluded from the regression models (< 0.1% missing). Eight people were missing age (0.6% missing) and were excluded from regression models. Due to variation in completeness of medical records, a subset of patients was missing complete information on time to appropriate treatment (n=75, 5.6% missing) or specific details on name, dose, or duration of antibiotic treatment (n=132, 9.9% missing). These patients were excluded from group comparisons of these variables by race. All group comparison tests were 2-sided and the test for time to treatment by race was 1-sided, as described in the manuscript. A p-value less than 0.05 was considered significant for all analyses.

Statistical analyses were performed using R (version 4.3.1, R foundation for Statistical Computing, Vienna, Austria) and SAS (version 9.4, SAS Institute Inc., Cary, NC, USA), and the graph was generated using GraphPad Prism (version 10.0.2, La Jolla, CA, USA).

© 2023 Starke SJ et al. *JAMA Network Open.*
