# Supplementary material for: Time to Diagnosis and Treatment of Lyme Disease by Patient Race
Source: JAMA Netw Open. 2023 Dec 12;6(12):e2347184. doi: 10.1001/jamanetworkopen.2023.47184 (PMC10716730; doi:10.1001/jamanetworkopen.2023.47184)
Supplement: Supplement 2. — Data Sharing Statement [file jamanetwopen-e2347184-s002.pdf]

Data Sharing Statement

Starke. Time to Diagnosis and Treatment of Lyme Disease by Patient Race. *JAMA Netw Open*. Published December 12, 2023. doi:10.1001/jamanetworkopen.2023.47184

Data
Data available: No
